# Acute Consumption of Blueberries and Short-Term Blueberry Supplementation Improve Glucose Management and Insulin Levels in Sedentary Subjects

**DOI:** 10.3390/nu13051458

**Published:** 2021-04-25

**Authors:** Ximena Palma, Samanta Thomas-Valdés, Gonzalo Cruz

**Affiliations:** 1 Centro de Investigación del Comportamiento Alimentario, Escuela de Nutrición y Dietética, Facultad de Farmacia, Universidad de Valparaíso, Valparaíso 2360102, Chile; ximena.palma@uv.cl; 2 Escuela de Nutrición y Dietética, Facultad de Farmacia, Universidad de Valparaíso, Valparaíso 2360102, Chile; 3 Laboratorio de Alteraciones Reproductivas y Metabólicas, Centro de Neurobiología y Fisiopatología Integrativa (CENFI), Instituto de Fisiología, Universidad de Valparaíso, Valparaíso 2360102, Chile

**Keywords:** blueberries, insulin, glucose management, antioxidant, polyphenols, sedentary subjects

## Abstract

Background: Blueberries are polyphenol-rich fruits with antioxidant and anti-inflammatory properties. Polyphenols from berries act by blocking digestive enzymes, reshaping gastrointestinal microbiota, and affecting the release of gastrointestinal hormones to regulate insulin dynamics and glucose management. However, most studies use fruit extracts instead of fresh fruit. We aimed to evaluate postprandial glucose management and antioxidant capacity of fresh blueberries consumed acutely or as a six-day supplementation in 10 sedentary subjects. Methods: To evaluate the effect of acute blueberry intake, 150 g of blueberries were consumed together with 150 g of white bread by the subject and blood samples were collected at 0, 30, 60, 90 and 120 min to measure glucose, insulin, and plasma antioxidant capacity. To evaluate supplementation, 150 g of blueberries were provided daily for six days and sample collection was performed at day 7. Results: Acute consumption of blueberries decreased postprandial glucose area under the curve (AUC) and increased insulin levels at 15 min timepoint. Supplementation did not affect glucose levels but decreased insulin levels at 120 min. No changes in antioxidant capacity were observed. Conclusions: Consumption of fresh blueberries improves postprandial glucose management presumably due to actions on the gastrointestinal tract, while supplementation improves insulin sensitivity, probably due antioxidant and anti-inflammatory effects.

## 1. Introduction

Type 2 diabetes (T2D) is a chronic disease characterized by progressive insulin resistance and subsequent hyperglycemia [1]. In 2017, more than 425 million people were estimated to present diabetes worldwide, and its prevalence is expected to increase by at least 50% by 2045 (WHO, 2016 World Health Organization, Global Report on Diabetes. WHO, 2016). This disorder depletes antioxidant defenses and induces oxidative stress, which enhance pancreatic beta cell damage and impairs appropriate insulin secretion [2]. Hyperglycemia promotes glucotoxicity that is distinguished by permanent oxidative stress resulting from generation of reactive oxygen and nitrogen species and/or changes in redox balance [3]. Glucotoxicity is associated with most macro- and microvascular complications occurring in T2D subjects [4].

Obesity and sedentarism are key independent risk factors for developing insulin resistance and other cardiometabolic disturbances [5]. Its deleterious impact has been highlighted by the confinement due to the COVID-19 pandemic [6]. In Chile, the last National Health Survey showed that 87% of adult subjects were classified as sedentary [7] and despite worldwide data being variable, physical inactivity reaches up to 80% in some subpopulations [8]. Therefore, it is necessary to modify lifestyle factors including physical activity and dietary interventions to preserve metabolic function and prevent T2D [9]. Consequently, important efforts have been made to identify alternative dietary compounds that could help in the management of insulin resistance and T2D. Polyphenols are natural compounds commonly found in fruits and vegetables, which have been studied for reduction of T2D risk [9,10]. Antioxidant and anti-inflammatory potential are ordinary attributes of polyphenols that could benefit T2D handling [10]. Estimated dietary intake of polyphenols indicate consumption as high as 1 g per day [11]. Berries contain a high content of polyphenols, ranging from 200 to 300 mg of per 100 g of fresh weight [12].

Some potential mechanisms by which polyphenols could influence glycemic control include: the inhibition of digestive enzymes, thus reducing glucose absorption [13]; gene modulation associated to β-cell dysfunction, insulin signaling pathways and gluconeogenesis pathways [14]; enhancement of insulin-mediated glucose uptake [15]; gut microbiota modulation [16]; improvement of pancreatic β-cell function [17] and liver glucose homeostasis [18]; and decreasing inflammatory pathways: [19]. Despite many possible mechanisms involving polyphenols’ effect on glycemic control, there have been limited successful clinical interventions using dietary polyphenols into glucose and insulin homeostasis [10], and most evidence relating polyphenols with glycemic control have used polyphenolic extracts or isolated compounds in their experimental design [17,18]). Many studies are also based on in vitro and animal assays [13,15,19]. The purpose of this study is to evaluate the effect of acute consumption of blueberries or six-day supplementation on glycemic and insulinemic responses and redox status after the ingestion of an available carbohydrate rich food in sedentary subjects.

## 2. Materials and Methods

### 2.1. Characterization of Blueberries

Blueberries were selected due to their antioxidant capacity and polyphenol content, especially anthocyanins. For this study, Jewel and Star varieties from Santa Adriana Farm (Quillota, Chile) were used. Fruits were collected, frozen at −20 °C and transported to the Nutrition Department at the Faculty of Medicine of the University of Chile, where they were stored at −20 °C to maintain their properties until analyses and interventions. Hydroalcoholic extract was prepared using methanol:water (80:20) to measure antioxidant capacity and polyphenol content. Antioxidant capacity was measured using the FRAP method described elsewhere by Benzie and Strain [20]. Briefly, the method involves the reduction of tripiridiltriazine (TPTZ)–Fe^+3^ complex at pH 3.6 that generates a blue color with a maximum wavelength at 593 nm. Results were expressed as mmol/Fe+^2^ per 100 g of fresh weight (mmol/ Fe+^2^/100 g FW). Similarly, polyphenol content was measured according to the Folin-Ciocalteau method previously described by Slinkard & Singleton, 1977 [21], based on the oxidation of phenol compounds. This reaction was measured by spectrophotometry at 765 nm and results were expressed as mg equivalent of gallic acid per 100 g of fresh weight (mg EGA/100 g FW). Total anthocyanin content was determined by the differential pH method described by Giusti & Wrolstad [22], an assay based on the molecular extinction coefficient of cyanidin 3-glucoside at different pH (1 and 4.5) and wavelengths (515 and 700 nm). The results were expressed as milligrams of equivalents of cyanidin 3-glycoside per 100 g of fresh weight (mg EC3G/100 g FW).

Portion size was determined as 150 g according to the definition of a regular edible portion found in the National Nutrient Database for Standard Reference Release 25 (USDA) to ensure subjects were able to easily eat the entire portion. It was instructed that each portion of blueberries should be eaten entirely, in the morning and without processing, and to keep them frozen until consumption to avoid losses due to processing and/or changes in storage temperature [23].

### 2.2. Subjects

Ten sedentary university students aged between 20 and 35 years old were recruited to participate in this study. Inclusion criteria considered body mass index (BMI) between 18.6 and 24.9 kg/m^2^; absence of non-communicable chronic disease, gastrointestinal or kidney pathologies; and being sedentary (less than 30 min of physical activity 3 times a week). Sedentary subjects were selected due to its high prevalence and to avoid the effect of physical activity over glycemic management [24], thus avoiding this acting as a confounding factor. Exclusion criteria were tobacco and alcohol consumption (over 400 mL a week); use of antihypertensive, hypoglycemic and/or lipid lowering drugs; antioxidant supplements consumption one month prior to the study; and pregnant or lactating in the case of female volunteers. Food Frequency Questionnaires (FFQ) were completed by the subjects to estimate dietary intake of fiber, vitamin C, vitamin E, carotenes and polyphenols previous to each intervention. FFQ analyses were based on the Chilean Food Composition Table and the USDA National Nutrient Database for Standard Reference. All subjects gave their written consent to participate in this study which was approved by the Faculty of Medicine Ethics Committee at the University of Chile.

### 2.3. Study Design

**Figure 1:**
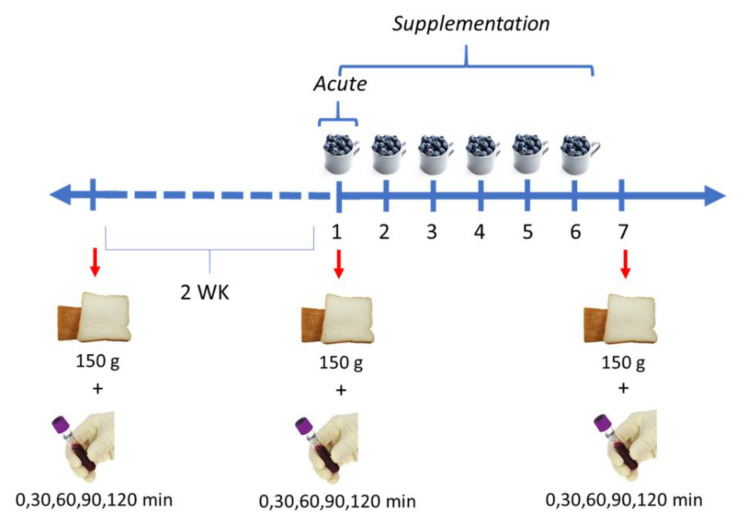
Design of the acute intervention and short-term supplementation. Acute intervention and short-term supplementation were separated by 2 weeks (WK).

Individuals acted as their own controls participating in an acute and a supplementation intervention. Subjects were scheduled at a minimum of two weeks between treatments to ensure an adequate washout period for the next intervention. For the baseline (control), subjects consumed 150 g of white bread. For the acute study, they consumed 150 g of white bread with 150 g of unprocessed frozen blueberries (immediately). For the short-term supplementation, subjects ate 150 g of unprocessed blueberries daily for 6 days, then they consumed 150 g of white bread at day 7 for blood collection (Figure 1).

### 2.4. Procedures

Participants were admitted to the laboratory on three separate occasions (control, acute intervention and short-term supplementation) after 10 h overnight fast. At 08:30 h, an intravenous cannula (BD Venflon, Oxford, UK) was inserted into an antecubital vein and a fasting blood sample was taken (time 0). At 09:00 h, subjects were given 150 g of white bread, equivalent to 75 g of available carbohydrates, according to nutritional information published by the producer (FUCHS, Santiago, Chile) and they consumed this within 15 min. Blood samples (5 mL) were drawn from the intravenous line at 30, 60, 90 and 120 min following the ingestion of bread. During the trial, the cannula was kept patent with regular 5 mL flushes of 0.9% NaCl saline (Baxter Healthcare, Northampton, UK) following each bloodletting. This procedure was repeated each time.

Blood samples were taken into heparinized tubes containing aprotinin (Sigma Chemical Laboratories Ltd., Poole, UK) for the analysis of insulin and into tubes containing sodium fluoride for glucose analysis. Samples collected for glucose were centrifuged immediately at 3000 rpm, 1000× *g* for 6 min and the plasma was separated, aliquoted and frozen at −20 °C until analysis. Additional samples were collected at 0, 30 and 120 min to determine plasma antioxidant capacity, total and oxidized glutathione (GSHt and GSSG, respectively), which were placed in tubes containing EDTA.

### 2.5. Chemical Analysis of Blood Samples

#### 2.5.1. Blood Glucose and Insulin

Plasma glucose concentrations were determined in duplicate by the GOD-PAP method, using a Human Pharma Latina kit. Insulin concentrations were measured using a chemiluminescence immunoassay. The incremental area under each 120 min plasma glucose and insulin response curve (AUC) were calculated using the trapezoidal rule with fasting values as the baseline (FAO/OMS, 1998).

#### 2.5.2. FRAP and Glutation

Plasma antioxidant capacity was measured using the FRAP method described above. The results were expressed as mg/ml. GSHt and GSSG concentration was measured in erythrocytes based on the method of Rahman et al. using 5,5-ditio-bis (nitrobenzoic acid), known as a DTNB reagent, to form 5-tio-2-nitrobenzoic acid observable by spectrophotometry at 405 nm. To determine concentration, the equation of the straight line of the measurements obtained for each sample (6 points) was calculated. With the resulting data, the net rate was defined by subtracting the value of the blank slope from each sample slope and the following formula was applied: GSHt = ((Net rate-intercept)/slope) * Dilution Factor, 0.0116 and 0.0012 being the slope and intercept values, respectively; the dilution factor was 90. GSHt concentrations were expressed in millimoles (mM) for 0, 30 and 120 min. GSSG measurements were carried out by reduction of GSSG to GSH in the presence of NADPH, and concentrations were determined using the same method as for GSHt, with 0.0197 and −0.002 the values of the GSSG slope and intercept, respectively. The corresponding dilution factor is 10.

### 2.6. Statistical Analysis

Data were expressed as median ± standard error. The Shapiro-Wilk test was used to confirm normality of the data. All variables were compared between acute blueberry consumption or supplementation and control (baseline) using two-tail paired *t*-test. A *p* value of less than 0.05 was considered significant. IBM SPSS statistics version 22.0 was used for FFQ analysis and GraphPad version 9.0 was used for other analyses.

## 3. Results

### 3.1. Blueberries’ Characteristics

Antioxidant capacity of blueberries was 2.56 ± 0.56 mmol Fe+^2^/100 g FW. Total polyphenols content and total anthocyanins were 256.02 ± 24.69 mg EGA/100 g FW and 63 ± 0.8 mg E C3G/100 g FW, respectively.

### 3.2. Participant Characteristics

Six women and four men, with a mean age of 26.3 ± 3.2 years and mean BMI of 22.59 ± 1.91 kg/m^2^ were recruited. Regarding basal metabolic parameters, glycemia was 91.19 ± 7.97 mg/dL in women and 95.04 ± 6.59 mg/dL in men, and insulinemia was 7.34 ± 4.17 and 6.54 ± 1.25 IU/mL in women and men, respectively. Regarding FFQ analyses, no statistical differences were observed when comparing the intake of fiber, vitamin C, vitamin E, carotenes and polyphenols prior to each treatment (Table 1).

### 3.3. Effects of Blueberry Consumption on Glucose Management

**Figure 2:**
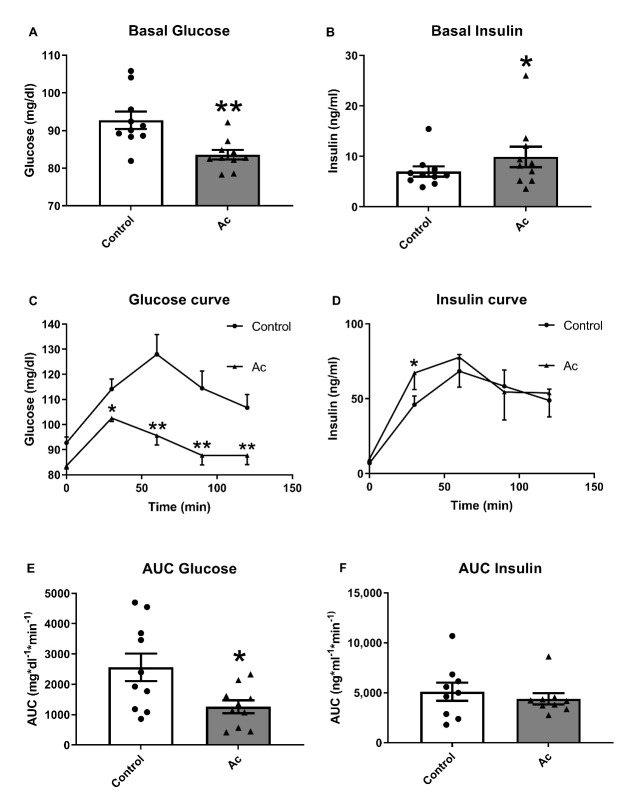
Glucose and insulin results after acute fresh blueberry consumption (Ac). (**A**) Basal fasting glucose levels. (**B**) Basal fasting insulin levels. (**C**) Glucose curve after 150 g of white bread consumption. (**D**) Insulin curve after 150 g white bread consumption. (**E**) Area under the curve (AUC) of plasma glucose after 150 g of white bread consumption. (**F**) Area under the curve (AUC) of plasma insulin after 150 g of white bread consumption. * *p* < 0.05 vs control or t = 0, ** *p* < 0.01 vs control or t = 0.

Figure 2 shows the effects of acute consumption of 75 g of carbohydrates, provided as white bread, with 150 g of blueberries on glucose and insulin levels compared to the levels previously determined in the same subjects without blueberry consumption (Control). Interestingly, acute blueberry consumption decreased glucose levels at 30, 60, 90 and 120 min after 75 g of carbohydrates intake, resulting in a significantly lower AUC. For insulin, concentration was higher only at 30 min when subjects had consumed 150 g of blueberries compared to control, but no differences were observed in AUC.

**Figure 3:**
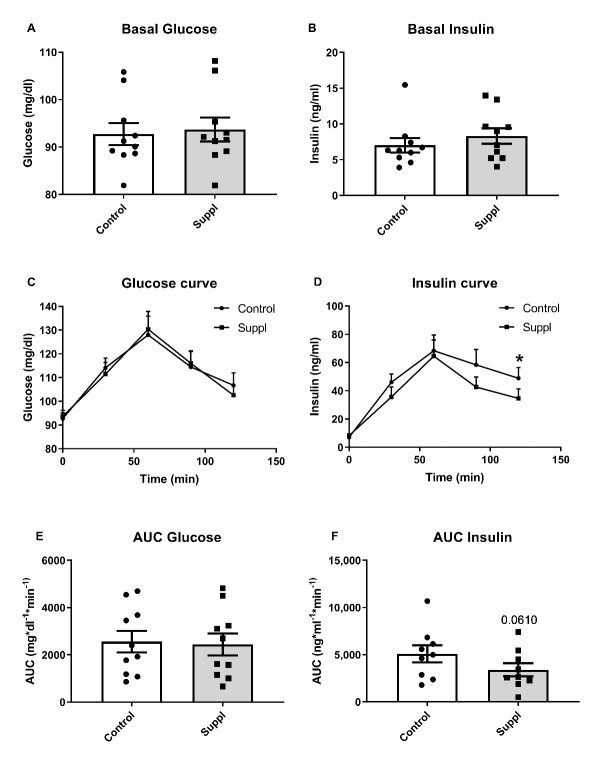
Glucose and insulin results after short-term fresh blueberry supplementation (Suppl). (**A**) Basal fasting glucose levels. (**B**) Basal fasting insulin levels. (**C**) Glucose curve after 150 g of white bread consumption. (**D**) Insulin curve after 150 g white bread consumption. (**E**) Area under the curve (AUC) of plasma glucose after 150 g of white bread consumption. (**F**) Area under the curve (AUC) of plasma insulin after 150 g of white bread consumption. * *p* < 0.05 vs. control.

Figure 3 shows the effects of 150 g of short-term supplementation of blueberries for six days on glucose and insulin concentration. Postprandial glucose levels were not affected while insulin levels were lower at 120 min. Insulin AUC showed a tendency to decrease compared to baseline.

### 3.4. Plasma Glutathione Levels

**Figure 4:**
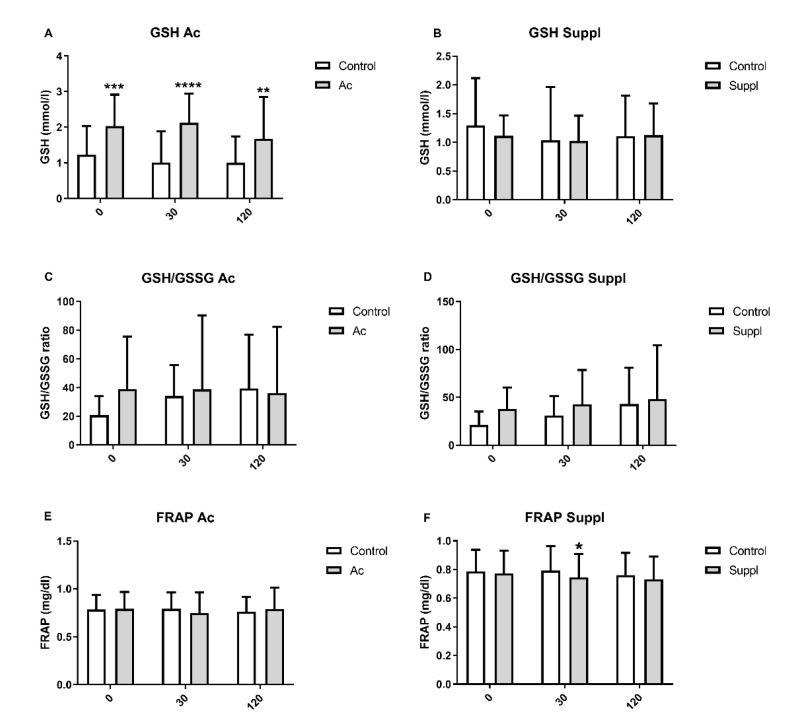
Plasma glutathione levels and antioxidant capacity measured by FRAP assay after acute consumption (Ac) and short-term blueberry supplementation (Suppl) compared to baseline levels (control). (**A**) GSH in acute consumption. (**B**) GSH in short-term supplementation. (**C**) GSH/GSSG in acute consumption. (**D**) GSH/GSSG in short-term supplementation. (**E**) FRAP in acute consumption. (**F**) FRAP in short-term supplementation. Baseline levels are shown by white bars while grey bars represent treatments. * *p* > 0.05 vs control, ** *p* < 0.01 vs control, *** *p* < 0.001 vs. control and **** *p* < 0.0001 vs. control. GSH: expressed as mM; GSSG: expressed as mM; FRAP: expressed as mg/dl.

Plasma glutathione levels were measured at 0, 30 and 120 min after consumption of 150 g of white bread in each of the interventions. Plasma GSH increased under acute consumption of 150 g of blueberries at the three timepoints, but short-term supplementation does not produce significant effects on GSH. GSH/GSSG was not affected in acute consumption or short-term supplementation with 150 g of blueberries (Figure 4).

### 3.5. Plasma Antioxidant Capacity

Plasma antioxidant capacity measured by FRAP at 0, 30 and 120 min was unchanged for both acute consumption of 150 g of blueberries and after six days of blueberries short-term supplementation (Figure 4).

## 4. Discussion

Blueberries are polyphenol-rich fruits with anti-inflammatory and antioxidant properties [25]. Most studies evaluating the effects of blueberries are performed in animal models using extracts or powder from fruits, but studies in humans are limited. In fact, few studies analyze the effects of blueberry fresh fruit on glucose management [26]. In the present study, we found that 150 g of consumption immediately improves significantly postprandial glucose levels (*p* < 0.05) and has beneficial effects on insulin sensitivity after a six-days supplementation in healthy subjects. A similar study with T2D individuals showed improvement of postprandial glucose response (glucose AUC) and reduction on insulin levels (insulin AUC) after acute consumption of blueberries containing 160 mg of anthocyanins followed by a 75 g glucose load [27].

The acute effects of fresh blueberries are probably mediated by their action on the gastrointestinal tract. Several polyphenols have been demonstrated to inhibit gastrointestinal enzymes, such as pancreatic α-amylase and α-glucosidase [13]. This inhibition could be the cause of lower glucose levels observed when blueberries were consumed with bread. However, insulin concentration did not decrease as expected. Instead, insulin significantly increased (*p* < 0.05) at 30 min after the intake of the bread portion. This could be explained by a possible insulinotropic effect of blueberry consumption. One of the most abundant polyphenols found in blueberries are anthocyanins that have been demonstrated to increase the release of intestinal insulinotropic peptides such as GLP-1 [28, 29]. However, a recent report demonstrated no effects of blueberries on GLP-1 and GIP levels in subjects consuming 140 g of blueberries once compared to placebo group [26]. The insulinotropic effect of blueberry consumption on glucose levels remains unclear and further research is needed.

Short-term blueberry supplementation did not affect postprandial glucose levels (*p* = 0.8414), but significantly decreased insulin levels at 120 min (*p* < 0.05) and tended to decrease insulin AUC (*p* = 0.0610). This could be the result of an improvement in insulin sensitivity, requiring lower insulin levels to regulate glucose concentrations. Similarly, after a supplementation of 45 g per day of freeze-dried blueberry for eight weeks, an improvement of insulin sensitivity of obese, nondiabetic, and insulin-resistant subjects was observed [30], while long-term blueberry supplementation (1 g of blueberry powder per day for 3 months) induced reduction of fasting and 2-h postprandial blood glucose, insulin levels and HOMA-IR in T2D volunteers compared to baseline results [31]. Despite these results, consumption of 22 g of freeze-dried blueberries for eight weeks did not change fasting plasma glucose and serum insulin in men with T2D [32]. The effect of blueberries on insulin sensitivity depends on different mechanisms which converge in decreasing inflammation.

The insulin sensitivity enhancement we had found specially in acute blueberry consumption could be partly explained by a significantly higher level of GSH when compared to control condition (*p* < 0.05). Oxidative stress and inflammation are associated with induces defects in glucose metabolism such as insulin-resistance. The improvement of antioxidant defense and control of inflammatory processes could help in the management of these defects [33]. In this sense, polyphenols have been largely studied as natural antioxidants using different models to prove their antioxidant and anti-inflammatory potential [34]. Regarding antioxidant defense, there is evidence that dietary polyphenols could regulate the expression of γ-glutamylcysteine synthetase, a rate-limiting enzyme present in the cellular GSH biosynthetic pathway [35]. Animal assays had found significantly higher GSH levels after administration of blueberry extract [36,37]. FRAP did not show any statistically significant difference in both acute intake and blueberry supplementation compared to control, except at 30 min for short-term supplementation (*p* < 0.05). In the same way, daily blueberry consumption (250 g) did not change the FRAP measurement in the plasma of chronic cigarette smokers after acute intake and three weeks supplementation [38]. Also, an acute consumption of 300 g of blueberries did not change oxidative stress and antioxidant defense in healthy man smokers [39]. However, an intervention using a blueberry drink for 6 weeks showed a significant reduc-tion of oxidized DNA bases and resistance to induce oxidative DNA damage [40].

Multiple inflammatory pathways are associated with glucose homeostasis and insulin response modulation. In vitro models using macrophages treated with LPS showed a significant reduction of reactive oxygen species after incubation with anthocyanins from blueberries [41]. According to the anti-inflammatory potential of blueberries, it was determined that blueberries’ anthocyanins reduced gene expression of IL-1β and TNF-α probably, due to declined translocation of NF-κB p65 to the nucleus [29,41]. Similarly, important inflammatory biomarkers, including mRNA of COX-2, IL-1β and iNOS, were inhibited by blueberry extract administration in murine RAW 264.7 macrophages [42]. A significant reduction of gene expression of TNF-α, IL-6, MCP-1 and iNOS associated with improvement of insulin sensitivity was observed in C57Bl/6j mice fed with a high fat diet supplemented with 4% blueberry powder for eight weeks [19]. The intestinal microbiota seems to be an important target to treat inflammation. Polyphenols are recognized by their prebiotic effect in modulation of the growth and activity of some colonic bacteria. This is because the polyphenols resist upper gastrointestinal digestion and reach the colon to be extensively metabolized by colonic microbiota [43]. Fecal Bifidobacteria levels were inversely associated with glucose intolerance and inflammatory biomarkers, including IL-1β, IL-6, TNF-α and MCP-1 [43,44,45]. Bifidobacterium spp. increased significantly after six weeks using a daily intake of a drink containing blueberry powder (25 g) compared to a placebo drink [16].

The present study has some considerations that are important to notice. First, our sample was composed of sedentary but healthy young participants, with a narrow range of BMI and age. We used one serving (150 g or 1 cup) of whole blueberries in both treatments, with the intention of testing a more feasible strategy. Our food approach takes into account the possible synergy between all compounds from the food matrix, which cannot be observed in extracts or supplements. A limitation of our study was the small sample size, which could have influenced the lack of statistical significance on the biomarkers measured and does not allow a deeper analysis of FFQ. Studies with a higher number of participants should be performed to determine if polyphenol-rich fresh fruits can be a part of the nutritional intervention for glucose management. Finally, herein we studied the effect of acute and short-term supplementation with fresh blueberries in sedentary subjects, but this scheme of blueberry consumption may have higher positive effects in patients with insulin resistance and T2D.

In conclusion, both acute blueberry consumption and short-term blueberry supplementation showed positive effects on glucose management and insulin homeostasis in sedentary subjects. Glucose concentrations were significantly lower after acute consumption, with no effect on insulin levels, presumably due to action on gastrointestinal enzymes inhibition and incretins secretion. Short-term supplementation showed a tendency to decrease insulin levels but not statistically significantly compared to control. Plasma GSH increased after acute consumption at the three timepoints, while GSH/GSSG and FRAP remained with no changes after blueberry consumption both acutely and by supplementation. Despite this, we strongly believe our study sets a good precedent regarding the use of whole blueberries as a dietary strategy to improve glucose homeostasis in sedentary young individuals.

## Figures and Tables

**Table 1:** Dietary intake of fiber and antioxidants in sedentary subjects on the day of acute consumption and supplementation experiments (Median ± SEM).

	Acute Consumption	Supplementation	*p*
Dietary fiber (g)	14.50 ± 2.59	18.17 ± 4.72	0.561
Vitamin C (mg)	150.74 ± 33.50	191.93 ± 35.33	0.562
Vitamin E (mg)	3.27 ± 0.56	5.20 ± 1.10	0.267
Carotenes (ug RE)	729.85 ± 205.07	1080.84 ± 261.15	0.472
Polyphenols (mg GAE)	401.23 ± 152.20	726.55 ± 256.64	0.469
